# Terretonin N: A New Meroterpenoid from *Nocardiopsis* sp.

**DOI:** 10.3390/molecules23020299

**Published:** 2018-01-31

**Authors:** Abdelaaty Hamed, Ahmed S. Abdel-Razek, Marcel Frese, Hans Georg Stammler, Atef F. El-Haddad, Tarek M. A. Ibrahim, Norbert Sewald, Mohamed Shaaban

**Affiliations:** 1 Organic and Bioorganic Chemistry, Faculty of Chemistry, Bielefeld University, D-33501 Bielefeld, Germany; abdohamed481@yahoo.com (A.H.); ahmedshukri_sci@yahoo.com (A.S.A.); marcel.frese@uni-bielefeld.de (M.F.); 2 Department of Chemistry, Faculty of Science, Al-Azhar University, Nasr City-Cairo 11884, Egypt; atefel_haddad@yahoo.com (A.F.E.-H.); tarekmostafaahmedibrahim@yahoo.com (T.M.A.I.); 3 Department of Microbial Chemistry, Division of Genetic Engineering and Biotechnology Research, National Research Centre, El-Buhouth St. 33, Dokki-Cairo 12622, Egypt; 4 Department of Chemistry, Inorganic and Structural Chemistry, Bielefeld University, D-33501 Bielefeld, Germany; georg.stammler@uni-bielefeld.de; 5 Department of Chemistry of Natural Compounds, Pharmaceutical and Drug Industries Research Division, National Research Centre, El-Buhouth St. 33, Dokki-Cairo 12622, Egypt

**Keywords:** *Nocardiopsis* sp. LGO5, terretonin N, 6-hydroxymeroterpenoid, antimicrobial activity, cytotoxicity

## Abstract

Terretonin N (**1**), a new highly oxygenated and unique tetracyclic 6-hydroxymeroterpenoid, was isolated together with seven known compounds from the ethyl acetate extract of a solid-state fermented culture of *Nocardiopsis* sp. Their structures were elucidated by spectroscopic analysis. The structure and absolute configuration of **1** were unambiguously determined by X-ray crystallography. The isolation and taxonomic characterization of *Nocardiopsis* sp. is reported. The antimicrobial activity and cytotoxicity of the strain extract and compound **1** were studied using different microorganisms and a cervix carcinoma cell line, respectively.

## 1. Introduction

The adaptation of marine bacteria to harsh environments has led to a rich biological and genetic diversity, so that structural diversity and complex molecular architectures became one of their key features of natural products with a remarkably broad range of biological activities [1,2,3,4,5,6,7]. Accordingly, they represent a potential source of new bioactive compounds for industrial, agricultural, environmental, pharmaceutical, and medical applications [8]. However, there are still many potential sources of new bioactive natural products from bacteria, as many habitats are yet unexplored [9].

Terpenes are an interesting class of bioactive compounds, which are produced biogenetically through either mevalonate or non-mevalonate pathways, comprising diverse skeletal structures (hemi-, mono-, sesqui-, di-, sester-, tri-, sesquart-, and tetraterpenes), and represent the largest group of natural products. They are native to plants (including macroalgae), invertebrates (sponges, soft corals), and fungi [10]. Recently, several terpenoidal and steroidal compounds with unique structures and activities were explored from actinobacteria, highlighting theses microorganisms as attractive sources of diverse bioactive compounds [11,12,13]. One such class of terpenes is known as sesterterpenoids and/or meroterpenoids, a relatively small group of terpenoids found in terrestrial plants, insects, fungi, lichens, and marine organisms [10,14,15,16,17]. They exhibit various bioactivities including antibacterial, antifungal, insecticidal, anti-inflammatory, cytotoxic, antifeedant, and antiaggregatory effects [18,19,20,21,22,23].

**Figure 1:**
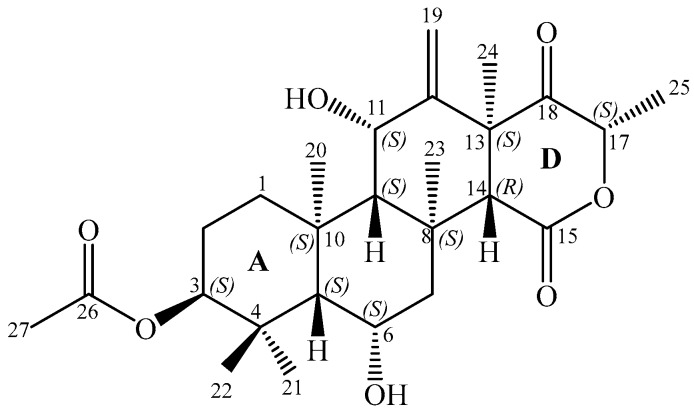
Structure of terretonin N (**1**).

In the course of our studies, one new highly oxygenated tetracyclic 6-hydroxymeroterpenoid, terretonin N (**1**) (Figure 1), was isolated from the ethyl acetate extract of a culture of *Nocardiopsis* sp. LGO5 fermented on solid medium. At the same time, the known compounds anthranilic acid, 3-indolylacetic acid, *N*^β^-acetyltryptamine [24], (3*R*,4*R*)-3,4-dihydroxy-3-methyl-pentan-2-one, (3*S*,4*R*)-3,4-dihydroxy-3-methyl-pentan-2-one [7], sitosteryl-3β-d-glucoside [25], and terrein [26,27] were obtained. The structure of **1** was elucidated using different analytical methods, and its absolute stereochemistry was unambiguosly determined by X-ray crystallography. Details of the isolation, taxonomical characterisation, and bioactivity of compound **1** are presented as well.

## 2. Results and Discussion

### 2.1. Isolation and Maintenance of the Producing Strain

The strain *Nocardiopsis* sp. LGO5 was isolated from a sediment sample collected from a lake in Helwan, Egypt. The strain was cultivated on starch-nitrate medium containing 50% natural sea water and incubated at 30 °C. The pure culture was maintained in glycerol at −80 °C and deposited at the Microbial Chemistry Department, National Research Centre, Cairo, Egypt.

### 2.2. Phenotypic and Genotypic Characteristics

**Figure 2:**
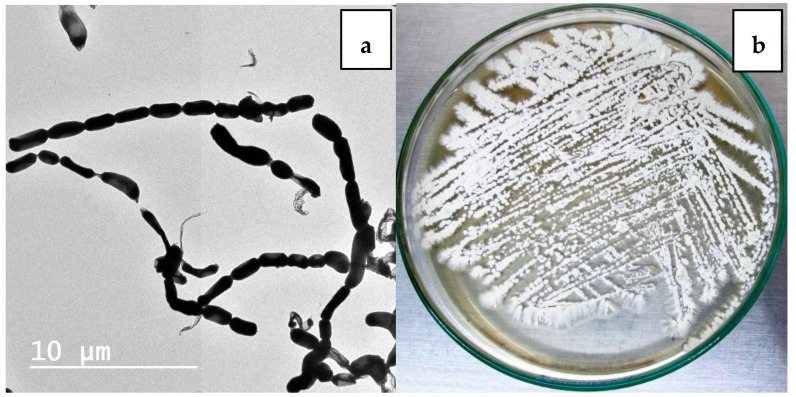
(**a**) Transmission electron micrography of strain showing elongated spores with smooth surface (bar = 10 µm); (**b**) 10-days age of LGO5 culture on yeast extract malt extract agar.

**Figure 3:**
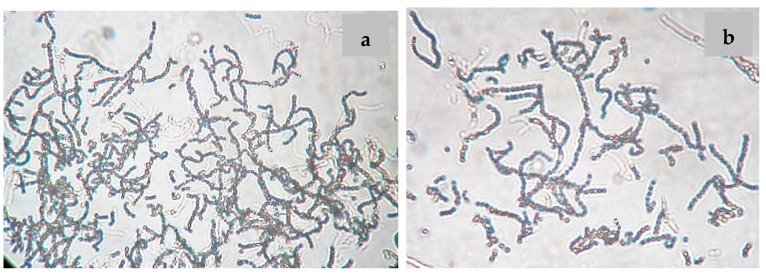
Spore chain morphology of strain LGO5 grown on starch-nitrate agar. Light microscopy of sporulating mycelium (1200×) showing long spiral to zig-zag spore chains: (**a**) in absence of nystatin; (**b**) in presence of nystatin.

*Nocardiopsis* sp. LGO5 forms a well-developed white aerial mycelium that turns into grey after 21 days, producing pale brown soluble pigments on starch nitrate agar and inorganic salt agar, whereas no melanoid pigments are formed on either ISP media 6 or 7. The strain shows white aerial mycelium on yeast extract-malt extract agar (Figure 2b). Cellobiose, galactose, glucose, maltose, and xylose, resp., were used as sole carbon sources. The strain grows in the presence of 10% (*w*/*v*) NaCl at 30 °C, 37 °C and 40 °C. Microscopically, the spores of LGO5 appear as long chains elongated to open *spirals* or *zig-zags* with a smooth surface (Figure 2a and Figure 3).

**Figure 4:**
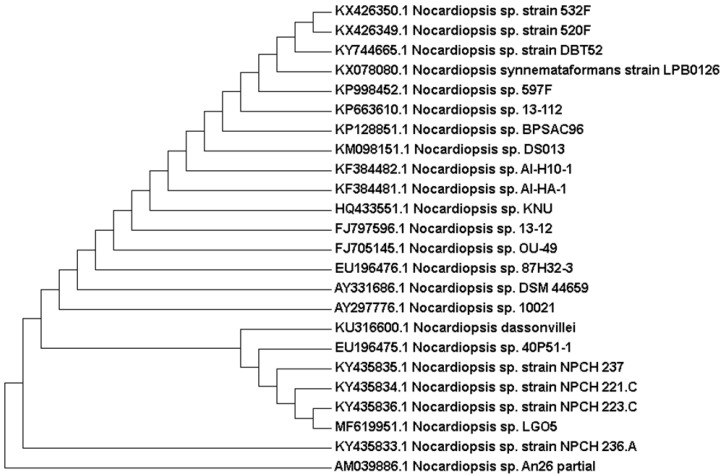
Evolutionary relationships of taxa related to *Nocardiopsis* sp. LGO5 based on 16S rRNA gene sequence using the Neighbour-Joining method. The analysis involved 24 nucleotide sequences.

**Figure 5:**
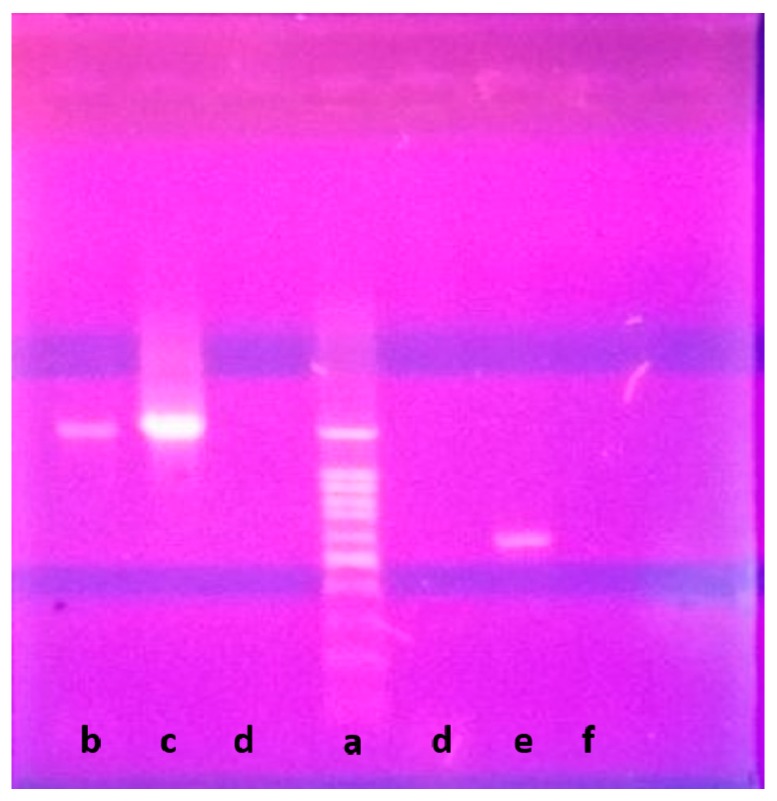
Agarose gel electrophoresis of LGO5 genomic DNA: (**Lane a**) DNA ladder 100 bp; (**Lane b**) PCR product of LGO5 genomic DNA using bacteria-specific primer; (**Lane c**) Positive control sample for known bacterial genomic DNA; (**Lane d**) Negative control sample using water instead of genomic DNA; (**Lane e**) Positive control sample for known fungal genomic DNA; (**Lane f**) PCR product of LGO5 genomic DNA using fungi-specific primer.

Genetically, 1270 bp of the 16S rRNA gene sequence was amplified from a pure culture of the strain LGO5 using PCR, sequenced, and analyzed using a BLAST-based approach. Phylogenetic analysis of this 16S rRNA gene sequence confirmed a very close relationship of the isolate LGO5 to *Nocardiopsis* spp. (Figure 4). The evolutionary history was inferred using the neighbour-joining method (Saitou and Nei, 1987) [28], and the evolutionary analyses were conducted in MEGA7 according to Kumar et al. 2016 [29]. Based on the mentioned characteristics, it was confirmed that the strain LGO5 belongs to *Nocardiopsis* sp., and it has been recorded in the GenBank database (accession No. MF619951). Additionally, the genomic DNA of LGO5 has been tested twice in PCR analysis using either a bacteria-specific primer or a fungi-specific primer. The results showed a specific band in the PCR product using the bacteria-specific primer (Figure 5b). Contrarily, the PCR product using the fungal primer did not show any band on agarose gel electrophoresis (Figure 5f).

### 2.3. Structure Elucidation

Chemical screening, monitored by thin-layer chromatography (TLC), of the bacterial extract exhibited several bands with a wide range of polarities. Non-UV-absorbing compounds were detected as intensive pink to violet bands with anisaldehyde/sulfuric acid. UV absorbing bands exhibited pink, yellow, or brown coloration with anisaldehyde/sulfuric acid. Separation of the produced metabolites by the strain was carried out using a series of chromatographic techniques. The physicochemical properties of terretonin N (**1**) are listed in Table 1.

Compound **1**, a moderately polar colourless solid, appeared on TLC as an intensive pink spot that changed to violet on spraying with anisaldehyde/sulfuric acid and heating. As it did not show UV absorption or fluorescence, characteristic for conjugated systems, we supposed it to be a terpenoid [30]. The molecular formula of **1** was determined as C_26_H_38_O_7_ by negative mode ESI-HR-MS (Table 1), indicating the presence of eight double bond equivalents (DBE).

The ^1^H-NMR spectrum together with HSQC data (Table 2) exhibited six methyl singlets at δ_H_ 2.03 (s, H_3_-23), 1.96 (s, H_3_-27), 1.68 (s, H_3_-20), 1.62 (s, H_3_-24), 1.28 (s, H_3_-22), 0.86 (s, H_3_-21), and one methyl doublet at δ_H_ 1.38 (d, 6.4 Hz, H-25). An oxymethine group resonating at δ_H_ 4.81 (m, H-17) was shown to be attached to a methyl group (H-25) based on the coupling constant (6.4 Hz) and H,H COSY crosspeaks. A pair of two singlets at δ_H_ 5.06 (s, H-19a) and 5.16 (s, H-19b) suggested a vinylidene moiety. Signals of further three oxymethine groups were detected; two of them are multiplet at δ_H_ 4.81 (H-11) and triplet (3.0 Hz) at δ_H_ 4.55 (H-3), while the third one resonated as triplet at δ_H_ 4.35 (H-6). A singlet of a non-oxygenated methine group δ_H_ 2.58 (s, H-14), as well as two doublets of doublets at δ_H_ 2.74 (dd, 14.5, 2.7 Hz, H-7a) and 1.19 (m, H-7b) corresponding to a methylene group, were detected. Further multiplet signals were detected at δ_H_ 2.08–0.95 with an integral of 6H, corresponding to two additional methylene groups (H_2_-1, H_2_-2) and two methine groups (H-5, H-9).

The ^13^C/DEPT/HSQC NMR spectra showed 26 resonance signals, corresponding to seven methyl groups (δ_C_ 24.2–14.6 [C-20–25,27]), three methylene groups (δ_C_ 50.3–22.5 [C-1,2,7]), and seven methine groups, among them four *sp*^3^ oxymethine groups (δ_C_ 79.4–76.0 [C-3,6,11,17]) and three non-oxygenated ones (δ_C_ 61.3–51.5 [C-5,9,14]). Nine quaternary carbons were found, among them one ketone carbonyl (206.8, C-18), two ester carbonyls of an α-lactone (δ_C_ 169.4, C-15) and an acetate residue (δ_C_ 170.6, C-26), and two *sp*^2^ carbons for an exocyclic double bond (δ_C_ 147.9 [C-12], 114.5 [C-19]). Further four quaternary carbons resonate in the aliphatic region (δ_C_ 50.0–37.1 [C-4,8,10,13]). The evidence mentioned above and the eight double bond equivalents deduced from the molecular formula suggested that compound **1** is a tetracyclic compound.

**Figure 6:**
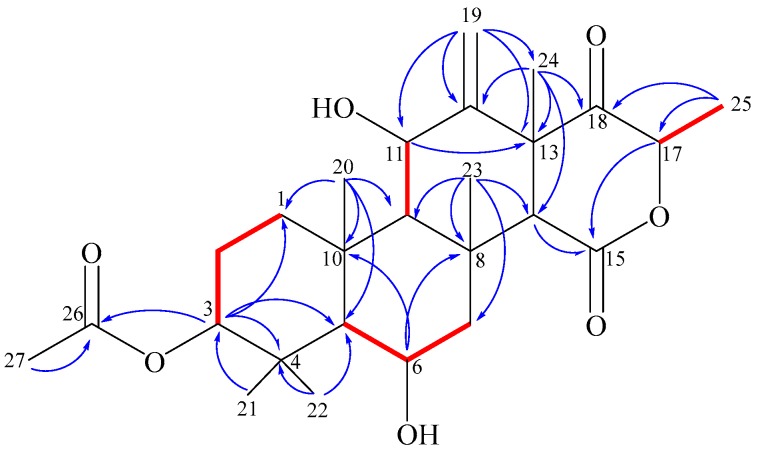
COSY (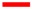) and key HMBC (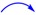) correlations of terretonin N (**1**).

According to H,H-COSY and HMBC experiments (Figure 6), compound **1** shows the following structural features: In ring A, the acetate residue is connected to the hydroxyl group at position 3 due to a visible ^3^*J* HMBC correlation between H-3 and C-26 (170.6). The *gem*-methyls (C-21,22) are attached to C-4. In ring B, the clear HMBC correlations from H-6 (4.35) to C-10 (38.1) and C-8 (37.1), and the H,H-COSY crosspeaks between H-6 and both neighboring protons (H-5 [1.25] and H-7 [2.74, 1.19]), completed the assignment. The HMBC correlations between H_3_-20 (1.68) and carbons C-1, 5, 9, and 10 established the fusion between rings A and B via C-5 and C-10. Alternatively, the direct fusion between rings B and C was proven through carbons C-8 and C-9 as the existence of an HMBC correlation from H_3_-23 (2.03) versus carbons C-7,8,9, and 14. The assignment of the vinylidene moiety at C-12 is based on the ^3^*J* HMBC correlations of corresponding methylene protons (H-19a/19b [5.06, 5.16]) with C-11 and C-13. The connectivity between rings C and D through C-13 and C-14 was verified by the HMBC correlation directed from the methyl H_3_-24 (1.62) towards C-12, 13, 14 and 18. The keto-lactone of ring D was identified by the ^3^*J* HMBC crosspeak between H-17 (4.81) and the lactone carbonyl C-15 (169.4), while the doublet methyl H_3_-25 (1.38) exhibited a ^3^*J* correlation towards the ketone carbonyl C-18 (206.8). According to the chromatographic behavior and based on the spectroscopic and mass spectrometric analyses together with data base searches, structure **1** was confirmed as a new meroterpenoidal/sesterterpenoidal compound and was given the trivial name terretonin N.

**Figure 7:**
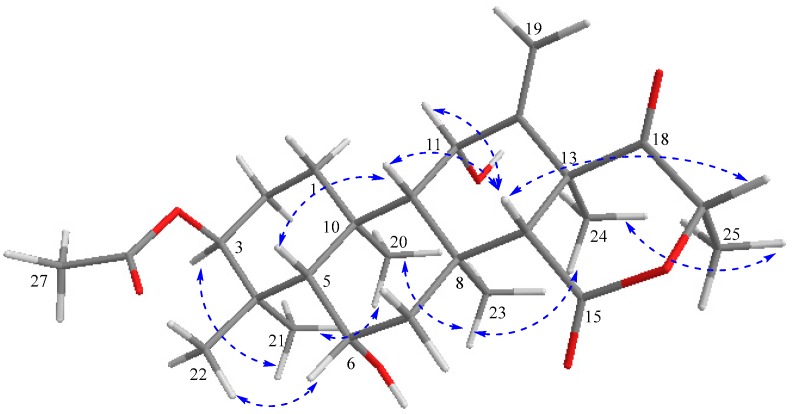
Key NOESY (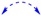) correlations of terretonin N (**1**).

**Figure 8:**
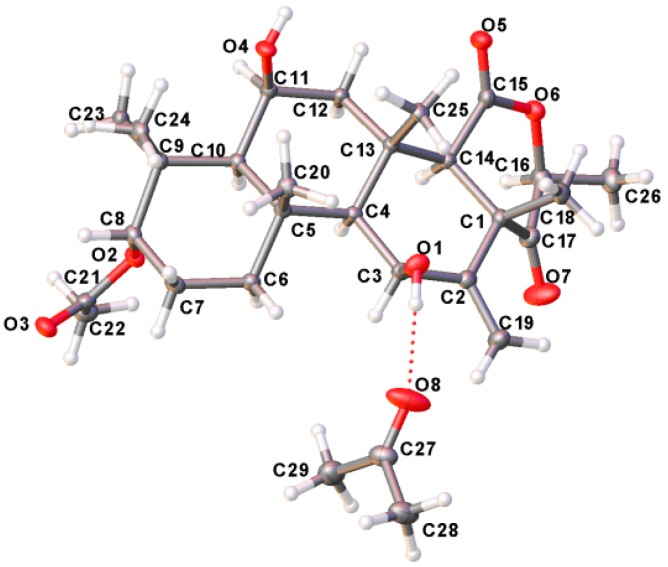
X-ray single crystal of terretonin N (**1**).

The relative stereochemistry of compound **1** was deduced from NOESY cross-peaks: H-3, CH_3_-21, CH_3_-20, CH_3_-23, CH_3_-24, and CH_3_-25 are located on one side of the molecule, while H-5, H-9, H-14, H-11, H-17, and CH_3_-22 point to the opposite side (Figure 7). Furthermore, the coupling constant of H-3 (*J* ~ 3.0 Hz) is indicative for its equatorial configuration, and, hence, the acetoxy residue has an axial orientation. The absolute configuration of terretonin N was established by X-ray structure determination (anomalous dispersion) of a single crystal grown from an acetone solution. The chiral carbons C-3, C-5, C-6, C-8, C-9, C-10, C-11, C-13, and C-17 have (*S*) configuration, while C-14 is the only stereocenter that has an (*R*) configuration (Figure 8).

Since the discovery of terpenes (more than 150 years ago), researchers have reported approximately 50,000 different terpenes derived from plants and fungi. Bacteria and other microorganisms are known to produce terpenes as well. However, they have received much less attention [31,32]. Cane et al. (Brown University, Providence, RI, USA) have proven the genetic capacity of bacteria to create terpenes with structural diversity [33,34,35,36]. During their studies 15 years ago, they have concluded, through the genome data gathered from many *Streptomyces* spp., that the latter have gene sequences that are similar to those encoding terpene synthases in plants and fungi. Consequently, they proved that actinobacteria indeed have genes for terpene synthases and that those enzymes could be used to make terpenes [37,38].

Terretonins are meroterpenoids biosynthetically derived from 3,5-dimethylorsellinic acid (DMOA), which is cyclized by terpene cyclase to form a terpenoid [39]. Recently, it has been reported that bacteria harbor numerous genes coding for terpene cyclases [40], in addition to terpene synthases, which are widely distributed in bacteria as well [11]. This underscores the eminent capability of bacteria to produce terpenoids with high similarity to those obtained from fungi. For example, merochlorin A, a sesterterpenoid, has been isolated from *Streptomyces* sp. [41]. Further intensive search in the literature and in the National Center for Biotechnology Information (NCBI) database (https://www.ncbi.nlm.nih.gov/protein/) revealed that thousands of gene cluster homologs encoding enzymes proposed to be involved in terretonin biosynthesis [42] have been identified in actinobacteria. These enzymes include terpene synthase and terpene cyclase, aromatic prenyltransferase, monooxygenase, polyketide synthases, methyltransferase, phytanoyl-CoA dioxygenase, epoxidase, cytochrome P450 monooxygenases, and short chain dehydrogenases. Such kinds of enzymes commonly exist in the bacterial systems as well. Additional studies and approaches are required to support the production of terretonins from bacteria, including the study of the full bacterial genome to characterize the gene cluster for terretonin biosynthesis.

### 2.4. Biological Activity

The crude extract of *Nocardiopsis* sp. LGO5 was tested against a set of microorganisms using the agar diffusion technique (Table 3), showing potent activity against *P. aeruginosa* and *S. cerevisiae*; (13 and 14 mm, respectively). It exhibited low to moderate activity against *E. coli* DSMZ 1058, *B. subtilis* DSMZ 704, *P. agarici* DSMZ 11810, *M. luteus* DSMZ 1605, *S. warneri* DSMZ 20036, *S. aureus, and C. albicans*. The pure compound **1** displayed pronounced activity in the antibacterial assay against the Gram positive *S. warneri* (zone of inhibition 15 mm), which even exceeds that of the positive control gentamycin (14 mm) (Table 4). However, it showed only low activity (7–8 mm) against the Gram negative *P. agaraci* and *E. coli*.

The bacterial extract and the purified compound terretonin N (**1**) did not exhibit any significant in vitro cytotoxicity against the human cervix carcinoma cell line KB-3-1.

## 3. Materials and Methods

### 3.1. General Experimental Details

Column chromatography was carried out on silica gel (0.06–0.2 mm, Merck, Darmstadt, Germany) deactivated with 3% aq. oxalic acid. Gel filtration was carried out on Sephadex LH-20 (GE Healthcare, Uppsala, Sweden). Preparative TLC (0.5 mm thick) and analytical TLC was performed on Merck pre-coated silica gel 60 PF_254+366_ plates (Merck, Darmstadt, Germany). *R*_f_ values of the bioactive compounds and visualization of their chromatograms was carried out under UV light (254 and 366 nm) and further by spraying with anisaldehyde/sulfuric acid followed by heating. High Resolution ESI-MS was done on a Micromass AC-TOF micro mass spectrometer (Micromass, Agilent Technologies 1200 series, Waldbronn, Germany). Optical rotations were measured on a P-1020 polarimeter (JASCO, Tokyo, Japan). 1D NMR and 2D (COSY, HMQC, HMBC, NOESY) NMR spectra were recorded on an Avance 500 MHz spectrometer (Bruker, Rheinstetten, Germany) at 500 MHz (^1^H) and 125 MHz (^13^C) at 298 K using the residual solvent peaks as a reference. X-ray crystallographic data of compound **1** was collected on a Rigaku Supernova diffractometer (Rigaku Innovative Technologies, Auburn Hills, MI, USA).

### 3.2. Isolation of Nocardiopsis sp. LGO5

The marine strain LGO5 was isolated from a sediment sample collected from a lake in Helwan, Egypt. It was cultivated on starch nitrate agar (20.0 g L^−1^ starch, 2.0 g L^−1^ KNO_3_, 1.0 g L^−1^ K_2_HPO_4_, 0.5 g L^−1^ MgSO_4_·7H_2_O, 0.5 g L^−1^ NaCl, 0.01 g L^−1^ FeSO_4_·7H_2_O, 3.0 g L^−1^ CaCO_3_, 20.0 g L^−1^ agar and 1000 mL of 50% sea water). The pH was adjusted to pH 7.2. The sediment sample was suspended into sterilized sea water and incubated for 30 min into a reciprocal water bath at 30 °C. The suspension was applied for a series of tenfold dilution with sterile sea water and platted (100 µL) on prepared media. The plates were then incubated at 30 °C for 6–8 weeks. Colonies with distinct morphological characteristics were selected and transferred onto freshly prepared solid media and stored in refrigerator at 4 °C. The strain LGO5 is deposited in the Microbial Chemistry Department, National Research Centre (NRC), Egypt.

### 3.3. DNA Isolation and 16S rRNA Gene Sequencing

The strain was inoculated in a 100 mL Erlenmeyer flask containing 50 mL of ISP2 medium (10 g L^−1^ malt extract, 4 g μL^−1^ yeast extract and 4 g L^−1^ glucose (pH 7.2) and incubated at 28 °C for 3 days. Genomic DNA of the strain was isolated using bacterial genome DNA isolation kit (Qiagen-kit: DNeasy Blood & Tissue Cat. No.: 69504) following the manufacturer’s manual.

The 16S rRNA gene was amplified from bacterial genomic DNA using primer set 9F (5′-GAGTTTGATCCTGGCTCAG3′)/1541R (5′-AAGGAGGTGATCCAACC3′). The following amplification profile was used: an initial denaturation step at 94 °C for 2 min was followed by 30 amplification cycles of 94 °C for 1 min, 55 °C for 1 min, 72 °C for 2 min, and a final extension step of 72 °C for 2 min. After agarose gel electrophoresis, the PCR product was detected and visualized by (UV) fluorescence after ethidium bromide staining [43]. PCR-clean-up was performed according to the description manual in Qiagen-kit: 28104. The 16S rRNA gene sequence was aligned using BLAST (blastn) available at NCBI database (National Centre Biotechnology Information, http://www.ncbi.nlm.nih.gov). The phylogenetic tree was constructed using neighbour-joining tree method using the software MEGA7. Further analyses for genomic DNA of LGO5 have been carried out using bead beating-extracted DNA and spin column purification by ABT DNA mini extraction kit (Applied Biotechnology Co. Ltd., Ismailia, Egypt). Briefly, the bacterial spores were suspended in 200 uL of distilled water containing zirconia silica beads, then homogenized by vortex mixing. Subsequently, the mixture was incubated at 100 °C for 15 min. Furthermore, the lysis buffer and proteinase K were added to the sample under investigation, and DNA extraction was preceded according to manufacturer instructions. The 16S rRNA gene was amplified using primer set (5′-AGAGTTTGATC(C/A)TGGCTCAG-3′)/(5′TACGG(**C**/**T**)TACCTTGTTACGACTT-3′). Additionally, the genomic DNA was further investigated using fungi-specific primer set ITS1 (5′-TCCGTAGGTGAACCTGCGG-3′)/ITS4 (5′-TCCTCCGCTTATTGATATGC-3′) to amplify 5.8S rRNA gene and ITS (Internal Transcript Spacer) regions (if exist). The following amplification profile was used: an initial denaturation step at 95 °C for 5 min was followed by 35 amplification cycles of 55 °C for 30 s, 72 °C for 90 s, and a final extension step at 72 °C for 3 min. The PCR products were detected and visualized by (UV) fluorescence after ethidium bromide staining.

### 3.4. Phenotypic Characterization of the Strain

The cultivation characteristics of the strain isolate LGO5 were studied according to the guidelines established in the International *Streptomyces* Project [17]. The aerial mycelium, pigmentation, and morphological features were examined after its growth on different ISP media. Physiological and biochemical characteristics were studied by NaCl tolerance [44], and different carbon sources utilization [17]. For microscopic examination, the bacterial strain has been cultivated on starch nitrate agar plates using two individual conditions: (a) in the presence of nystatin [100,000 IU/mL] (50 μL/500 mL media) as antifungal agent; and (b) in absence of the antifungal agent. The bacterial cells obtained during both were examined for spore chain morphology under light microscope (Olympus CH-2) using 40× lens (Figure 3). Electron micrograph has been taken using Transmission Electron Microscope (TEM) at National Research Centre (NRC), Egypt (Figure 4a)

### 3.5. Large-Scale Fermentation and Isolation

The spore suspension of strain LGO5 was inoculated into 100 mL of ISP2 medium and cultivated at 30 °C for 3 days as seed culture. 5 mL of seed culture were aseptically transferred to inoculate 1 L Erlenmeyer flasks (12 flasks) containing modified rice medium composition: 100 g commercial rice and 100 mL 50% sea water containing 0.4% yeast extract and 1% malt extract. The flasks were incubated for 14 days at 35 °C. After harvesting, the obtained broth was macerated in methanol (2.5 L). The methanol extract was separated by filtration, and concentrated *in vacuo*, and the remaining water residue was re-extracted with ethyl acetate. The obtained ethyl acetate extract was finally concentrated *in vacuo* to dryness and then further purified.

The crude extract (3.9 g) was separated by column chromatography on silica gel (60 × 3 cm) with a cyclohexane/dichloromethane (DCM)/methanol (MeOH) gradient, 0.5 L cyclohexane, 0.5 L cyclohexane/DCM [8:2], 0.5 L cyclohexane/DCM [6:4], 0.5 L cyclohexane/DCM [4:6], 0.5 L cyclohexane/DCM [2:8], 0.5 L DCM, 0.3 L DCM/MeOH [97:3], 0.3 L DCM/MeOH [95:5], 0.3 L DCM/MeOH [90:10], 0.3L DCM/MeOH [80:20], and 0.3 L MeOH. According to TLC monitoring, three fractions were obtained: FI (0.97 g), FII (1.2 g), and FIII (0.3 g). Fraction FI was highly nonpolar, mostly fats, and was discarded. Purification of FII on Sephadex LH-20 (CH_2_Cl_2_/40% MeOH) yielded anthranilic acid (1 mg) and indole-3-acetic acid (1.2 mg) in addition to four sub-fractions, FIIa, FIIb, FIIc, and FIId. Sub-fraction FIIa was purified using Sephadex LH-20 (CH_2_Cl_2_/40% MeOH) to afford terretonin N (**1**, 8 mg) as colorless needles. The diastereomers (3*R*,4*R*)-3,4-dihydroxy-3-methylpentan-2-one and (3*S*,4*R*)-3,4-dihydroxy-3-methylpentan-2-one (6 mg) were obtained as colourless oil from sub-fraction FIIb after its application to silica gel (column 60 × 1.5 cm) and elution with CH_2_Cl_2_/MeOH gradient. Further purification of sub-fraction FIIc on Sephadex LH-20 (CH_2_Cl_2_/40% MeOH) gave (2 mg) of tryptamine. The last sub-fraction FIId was purified on Sephadex LH-20 (CH_2_Cl_2_/40% MeOH) to afford terrein (4 mg). Finally, application of fraction FIII to Sephadex LH-20 (CH_2_Cl_2_/40% MeOH) afforded sitosteryl-3β-d-glucoside (3 mg) as colorless solid.

### 3.6. Antimicrobial Activity Assay

Antimicrobial activity testing of the bacterial crude extract and compound **1** was carried out against a set of microorganisms using paper-disk diffusion assay [45] with some modifications according to our previous work [7].

### 3.7. Cytotoxicity Assays

Methodology of the cytotoxic assaying of the bacterial extract and compound **1** against the human cervix carcinoma cells KB-3-1 was carried out according to our previous work [7].

### 3.8. Crystal Data

Terretonin N: C_29_H_44_O_8_ (Terretonin N, [C_26_H_38_O_7_] + acetone [C_3_H_6_O], N = 520.64 g/mol), monoclinic, space group P2_1_ (no. 4), *a* = 6.95341(3) Å, *b* = 20.21914(9) Å, *c* = 9.76794(5) Å, β = 97.2649(5)°, *V* = 1362.269(11) Å^3^, *Z* = 2, *T* = 100.0(1) K, μ(CuKα) = 0.744 mm^−1^, *Dcalc* = 1.269 g/cm^3^, 76,570 reflections measured (8.746° ≤ 2Θ ≤ 152.702°), 5690 unique (*R*_int_ = 0.0248, R_sigma_ = 0.0079), which were used in all calculations. The final *R*_1_ was 0.0221 for 5677 reflections with *I* > *2*σ*(I)* and *wR*_2_ was 0.0597 for all data, Flack parameter −0.01(2). For further details see the CIF file attached in the Supplementary Materials. CCDC 1575850 contains the supplementary crystallographic data for this paper. These data can be obtained free of charge via http://www.ccdc.cam.ac.uk/conts/retrieving.html.

## 4. Conclusions

A new, highly oxygenated, and unique tetracyclic 6-hydroxymeroterpenoid, terretonin N (**1**) was isolated from the marine-derived *Nocardiopsis* sp. LGO5 together with further seven known metabolites. The chemical structure of **1** was elucidated by extensive studies of 1D NMR, 2D NMR, and ESI HR MS data. The configuration of **1** was established by NOESY and X-ray crystallography. Terretonin N (**1**) exhibited high activity against Gram-positive bacteria with no cytotoxicity, reflecting its importance in pharmacology and pharmacognosy studies concerning drug discovery and its development.

## Figures and Tables

**Table 1:** Physico-chemical properties of terretonin N (**1**).

	1
Appearance	Colourless solid
*R*_f_ (Silica gel G/UV254, (CH_2_Cl_2_/5% MeOH))	0.54
Staining with anisaldehyde/ sulfuric acid	Pink and later as violet
Molecular formula	C_26_H_38_O_7_ (462)
(+)-ESI-MS: *m*/*z* (%)	485 ([M + Na]^+^, 100), 947 ([2M + Na]^+^, 0.58)
(−)-ESI-MS: *m*/*z* (%)	461 ([M − H]^−^, 62), 891 ([2M − H]^−^, 4)
(−)-HR-ESI-MS: *m*/*z*	
Found	461.2547 [M − H]^−^
Calcd.	461.2545 [M − H]^−^
[α]${}_{D}^{20}$	−114 (*c* = 0.1, MeOH)

**Table 2:** ^13^C (125 MHz) and ^1^H (500 MHz) NMR data of terretonin N (**1**) in CDCl_3_.

Position	δ_C_ [ppm]	δ_H_ [ppm] (m, *J* [Hz])	Position	δ_C_ [ppm]	δ_H_ [ppm] (m, *J* [Hz])
1	35.4	1.65 (m), 1.21 (m)	14	59.1	2.58 (s)
2	22.5	1.95 (m), 1.65 (m)	15	169.4	
3	79.4	4.55 (t, 3.0)	17	76.8	4.81 (m)
4	37.6		18	206.8	
5	51.5	1.25 (m)	19	114.5	5.16 (s), 5.06 (s)
6	68.1	4.35 (t, 3.0)	20	18.8	1.68 (s)
7	50.3	2.74 (dd, 14.5, 2.7), 1.19 (m)	21	27.8	0.86 (s)
8	37.1		22	23.7	1.28 (s)
9	61.3	0.95 (d, 2.4)	23	19.0	2.03 (s)
10	38.1		24	24.2	1.62 (s)
11	76.0	4.81 (m)	25	14.6	1.38 (d, 6.4)
12	147.9		26	170.6	
13	50.0		27	21.3	1.96 (s)

**Table 3:** Antimicrobial activities of LGO5 crude extract using agar diffusion test (mm diameter).

	EC ^a^	BS ^b^	Psa ^c^	Ml ^d^	Stw ^e^	Sta ^f^	Psae ^g^	Ca ^h^	Sac ^i^
LGO5 extract	7	8	9	11	8	10	13	7	14
Gentamycin	19	18	20	16	14	20	20	18	15

^a^ *E. coli* DSMZ 1058; ^b^ *Bacillus subtilis* DSMZ 704; ^c^ *Pseudomonas agarici* DSMZ 11810; ^d^ *Micrococcus luteus* DSMZ 1605; ^e^ *Staphylococcus warneri* DSMZ 20036; ^f^ *Staphylococcus aureus*; ^g^ *Pseudomonas aeruginosa*; ^h^ *Candida albicans*; ^i^ *Saccharomyces cerevisiae*.

**Table 4:** Antimicrobial activities of pure compounds using agar diffusion test (mm diameter).

	EC ^a^	BS ^b^	Psa ^c^	Ml ^d^	Stw ^e^
Terretonin N	8	–	7	–	15
Gentamycin	19	18	20	16	14

^a^ *E. coli* DSMZ 1058; ^b^ *Bacillus subtilis* DSMZ 704; ^c^ *Pseudomonas agarici* DSMZ 11810; ^d^ *Micrococcus luteus* DSMZ 1605; ^e^ *Staphylococcus warneri* DSMZ 20036, (–) = not active.

## Supplementary Materials

The supplementary data are available online.
