# Supplementary material for: Terretonin N: A New Meroterpenoid from Nocardiopsis sp
Source: Molecules. 2018 Jan 31;23(2):299. doi: 10.3390/molecules23020299 (PMC6017310; doi:10.3390/molecules23020299)
Supplement: Supplementary file 1 [file molecules-23-00299-s001.pdf]

Supplementary Materials

Terretonin N: a New Meroterpenoid from *Nocardiopsis* sp.

Abdelaaty Hamed[1,2], Ahmed S. Abdel-Razek[1,3], Marcel Frese[1], Hans Georg Stammler[4], Atef F. El-Haddad[2], Tarek M. A. Ibrahim [2], Norbert Sewald[1*], and Mohamed Shaaban[1,5*]

1 Organic and Bioorganic Chemistry, Faculty of Chemistry, Bielefeld University, D-33501 Bielefeld, Germany; abdohamed481@yahoo.com, ahmedshukri_sci@yahoo.com, marcel.frese@uni-bielefeld.de, norbert.sewald@uni-bielefeld.de , mshaaba@gmail.com

2 Chemistry Department, Faculty of Science, Al-Azhar University, Nasr City-Cairo 11884, Egypt; abdohamed481@yahoo.com, tarekmostafaahmedibrahim@yahoo.com, atefel_haddad@yahoo.com

3 Microbial Chemistry Department, Division of Genetic Engineering and Biotechnology Research, National Research Centre, El-Buhouth St. 33, Dokki-Cairo 12622, Egypt; ahmedshukri_sci@yahoo.com

4 Inorganic and Structural Chemistry, Bielefeld University, Department of Chemistry, D-33501 Bielefeld, Germany; georg.stammler@uni-bielefeld.de

5 Chemistry of Natural Compounds Department, Division of Pharmaceutical Industries, National Research Centre, El-Buhouth St. 33, Dokki-Cairo 12622, Egypt; mshaaba@gmail.com

* Correspondence:
norbert.sewald@uni-bielefeld.de; Tel.: +49-(0)521-106 2051
mshaaba@gmail.com; Tel: +202-2701728-1550

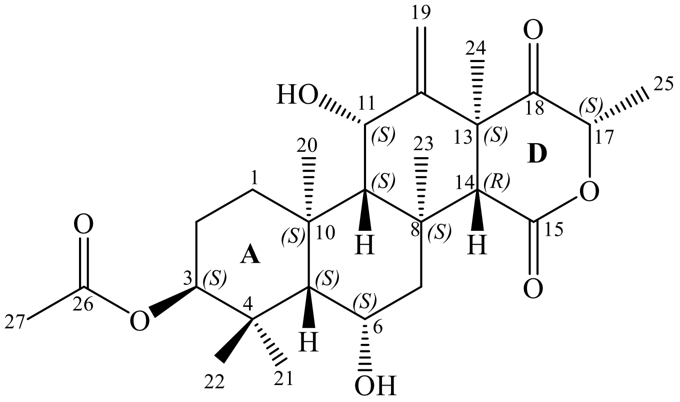

Figure S1. Structure of terretonin N (**1**)

**Table of Contents:**	**Page**
**Figure S1:** Structure of terretonin N (**1)**	S1
**Figure S2:** A comparison between the producing actinobacteria *Nocardiopsis* sp in a) Petri-dish for the original culture, and b) large scale fermented one, indicating their high identity (Chalky appearance whitish grey mycelia**)**	S3
**Figure S3:** (+)-ESIMS of terretonin N (**1**)	S4
**Figure S4:** (-)-ESIMS of terretonin N (**1**)	S5
**Figure S5:** (-)-HR-ESIMS of terretonin N (**1**)	S6
**Figure S6:** ^{1}H NMR spectrum ($CDCl_3$, 500 MHz) of terretonin N (**1**)	S7
**Figure S7:** ^{13}C NMR spectrum ($CDCl_3$, 125 MHz) of terretonin N (**1**)	S8
**Figure S8:** DEPT spectrum ($CDCl_3$, 125 MHz) of terretonin N (**1**)	S9
**Figure S9:** H,H-Cosy spectrum ($CDCl_3$, 500 MHz) of terretonin N (**1**)	S10
**Figure S10:** HMQC spectrum ($CDCl_3$, 500 MHz) of terretonin N (**1**)	S11
**Figure S11:** HMBC spectrum ($CDCl_3$, 500 MHz) of terretonin N (**1**)	S12
**Figure S12**: NOESY spectrum ($CDCl_3$, 500 MHz) of terretonin N (1)	S13

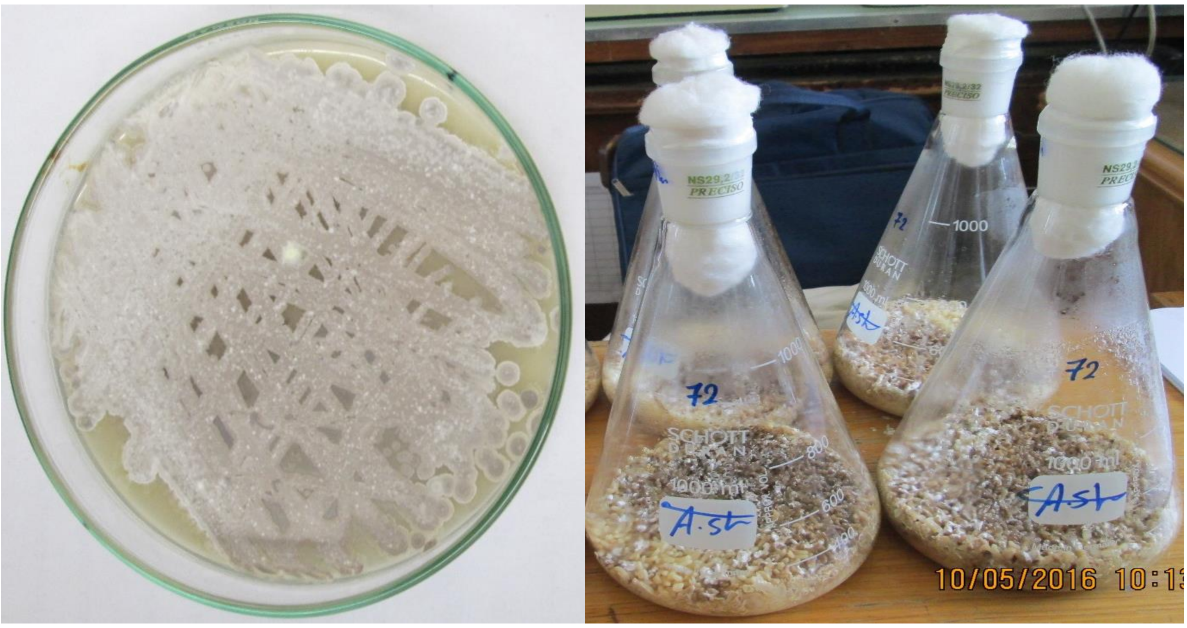

Figure S2: A comparison between the producing actinobacteria *Nocardiopsis* sp in a) Petri-dish for the original culture, and b) large scale fermented one, indicating their high identity (Chalky appearance whitish grey mycelia**)**

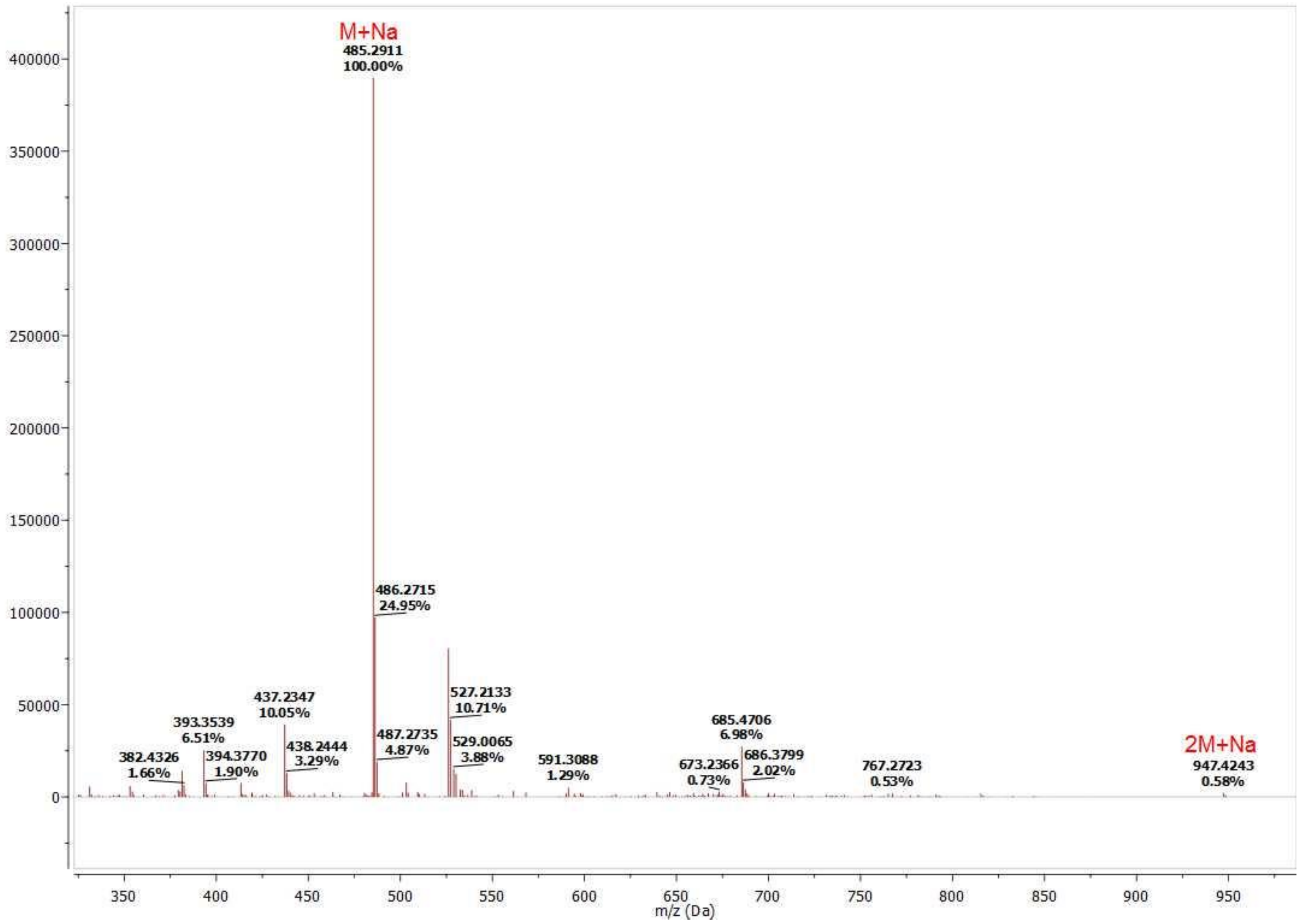

Figure S3: (+)-ESIMS of terretonin N (**1**)

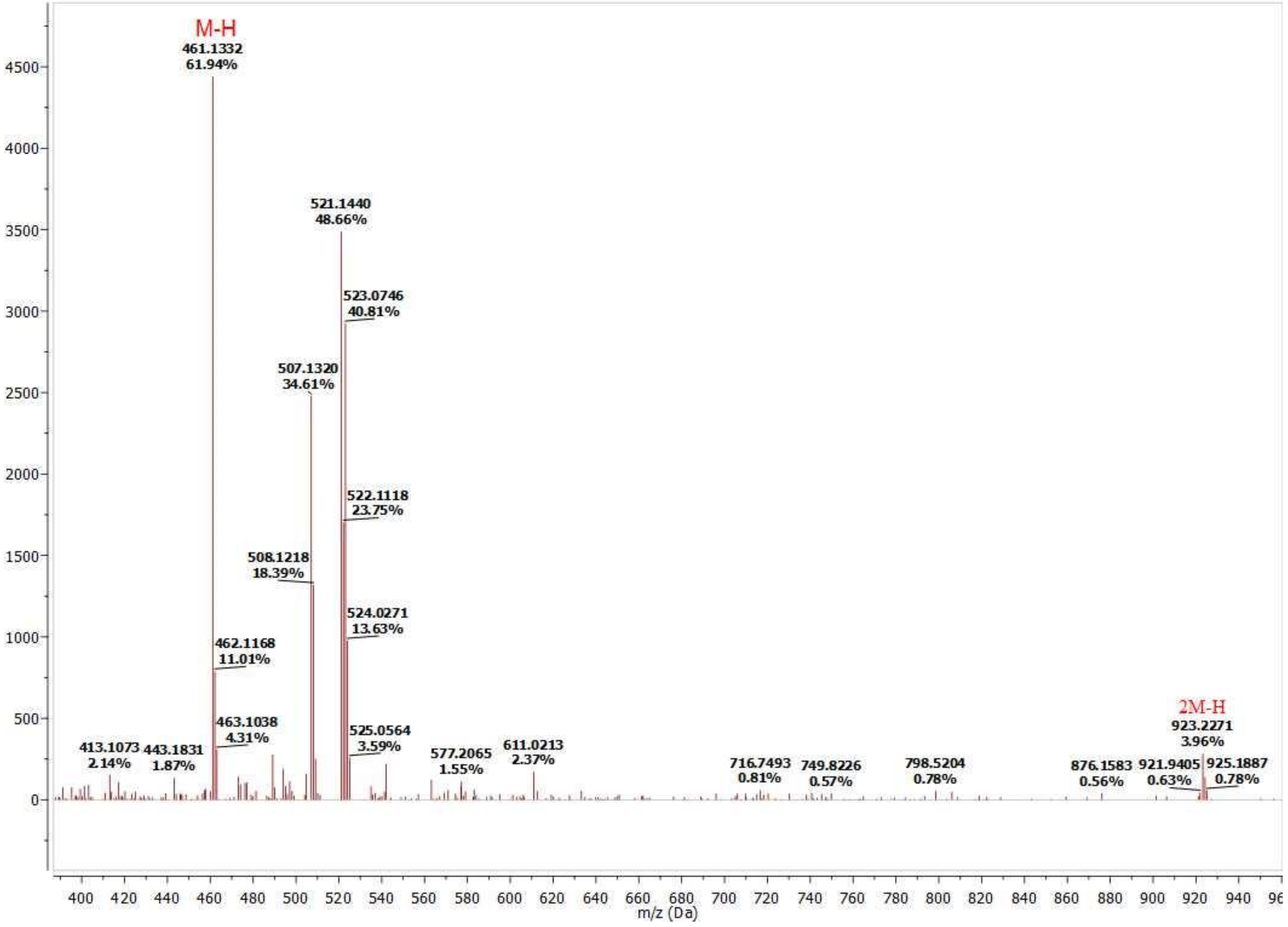

Figure S4: (-)-ESIMS of terretonin N (**1**)

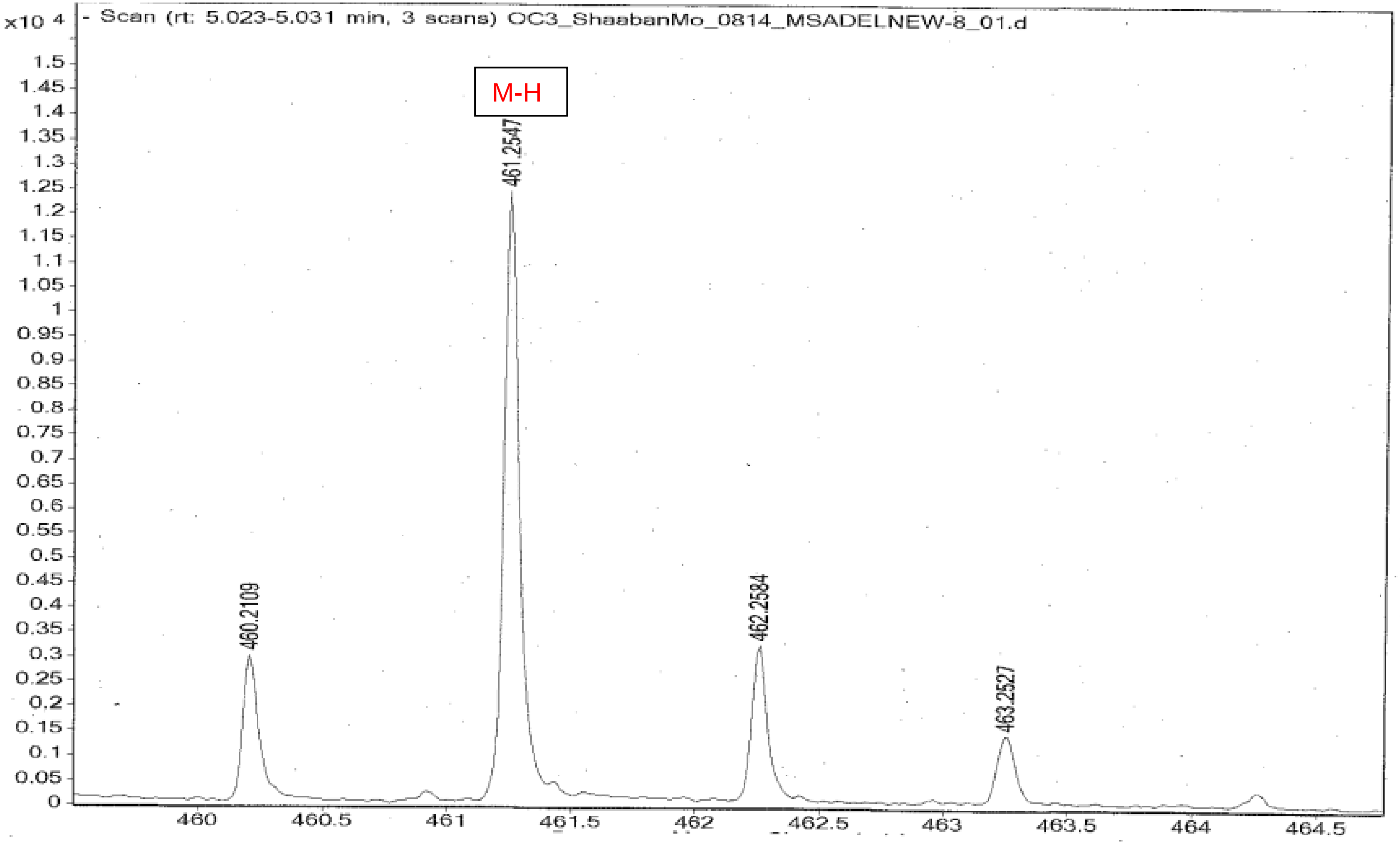

Figure S5: HR-ESI MS of terretonin N (**1**)

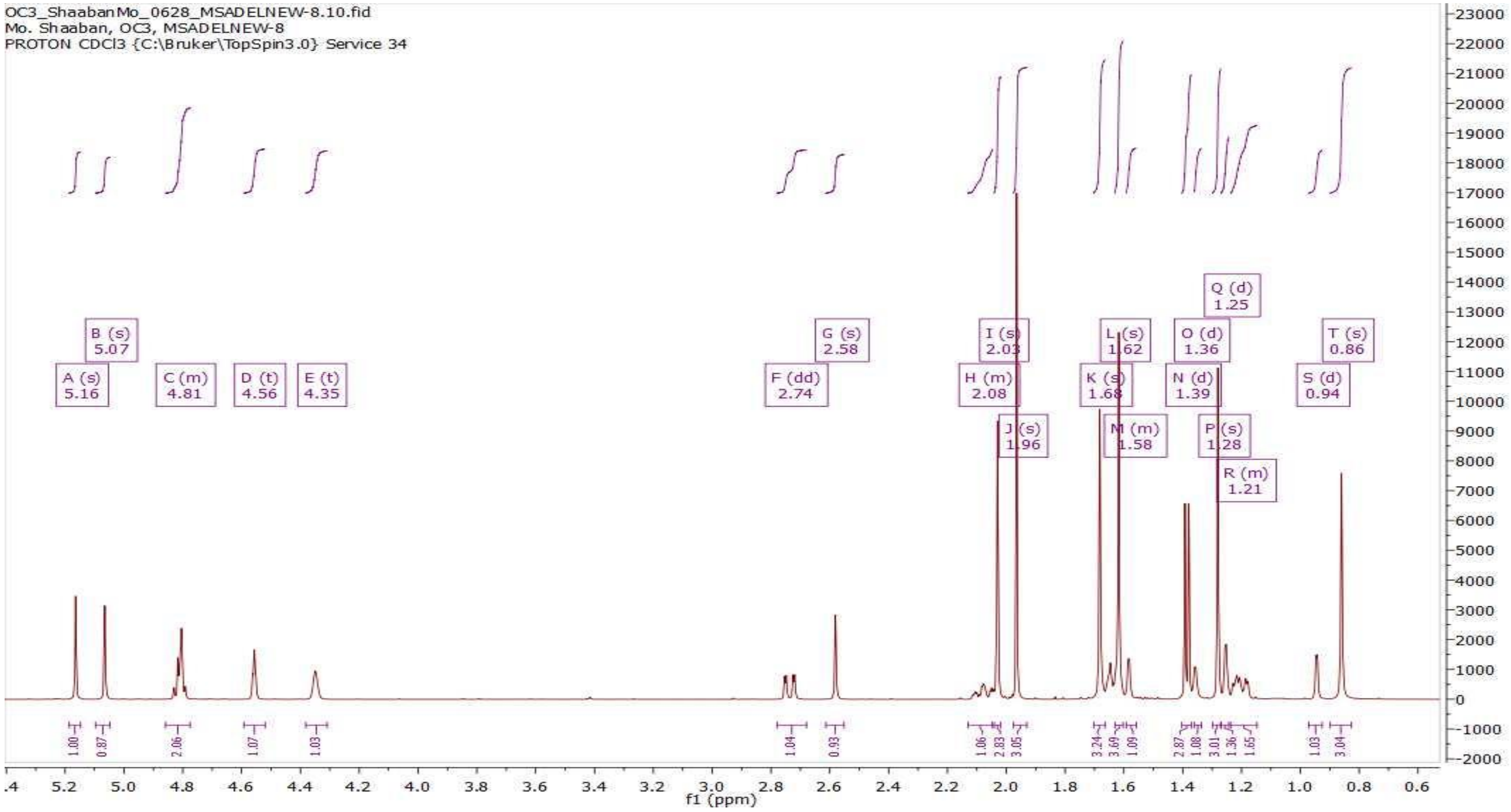

Figure S6: ^{1}H NMR spectrum ($CDCl_3$, 500 MHz) of terretonin N (**1**)

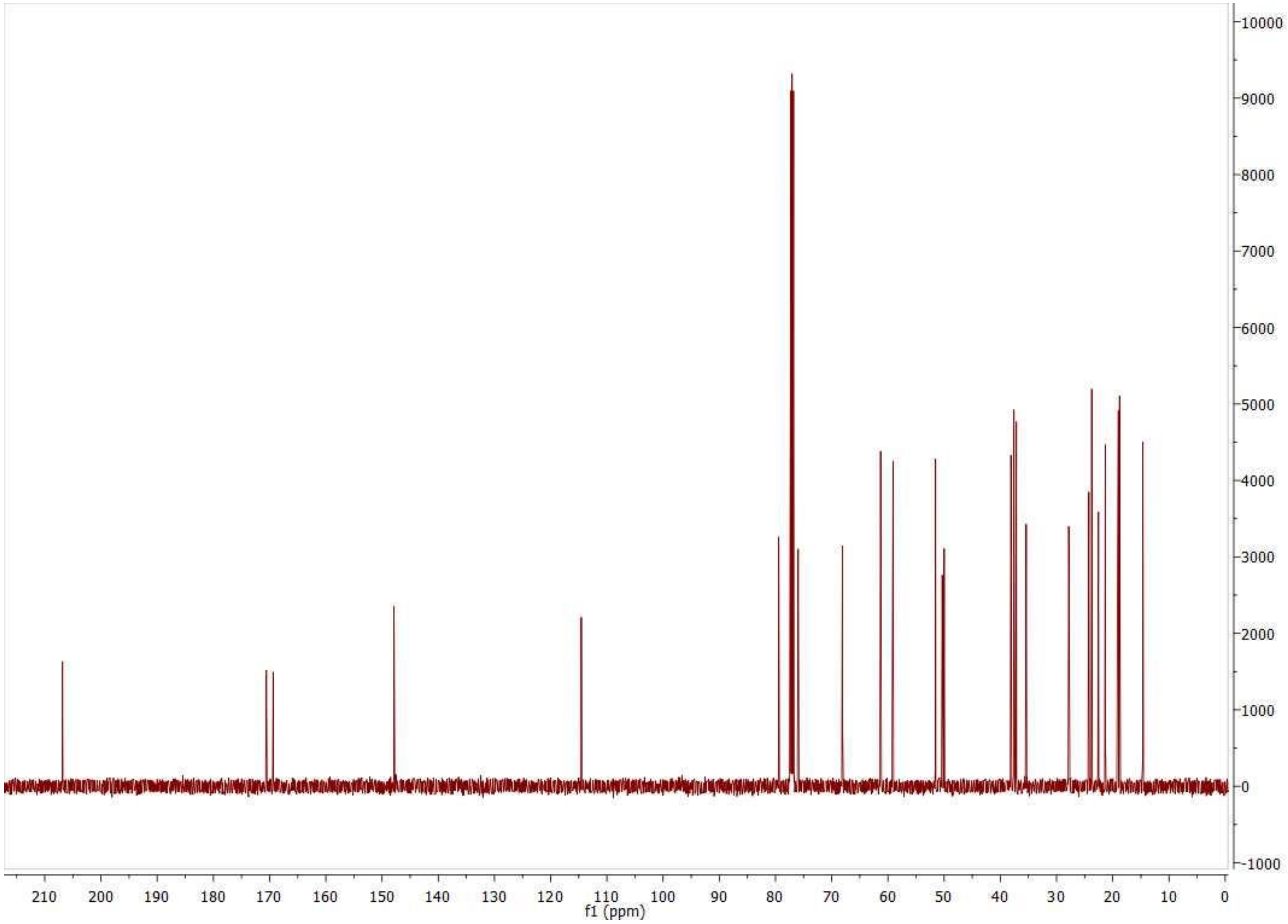

Figure S7: ^{13}C NMR spectrum ($CDCl_3$, 125MHz) of terretonin N (**1**)

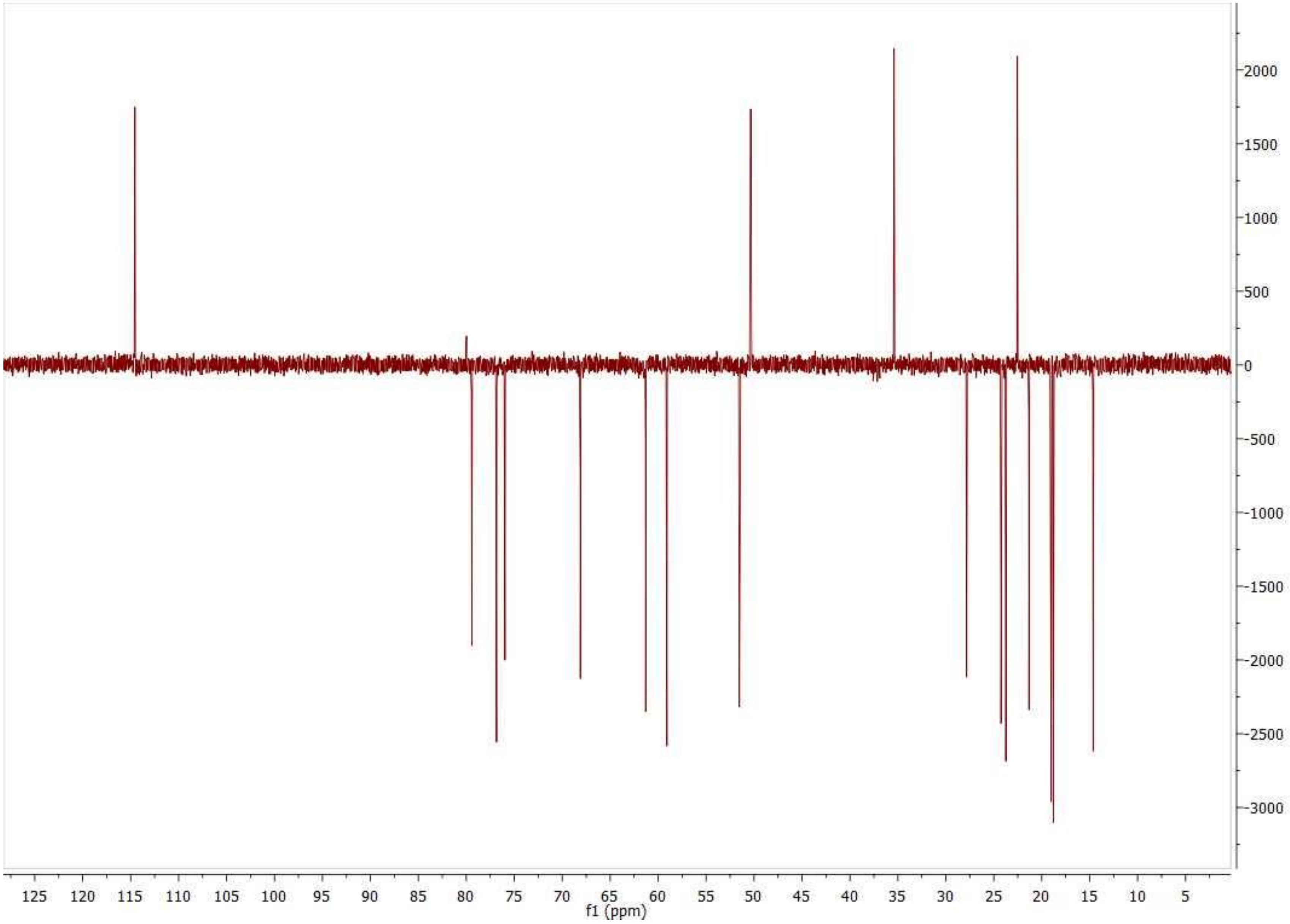

Figure S8: DEPT spectrum ($CDCl_3$, 125 MHz) of terretonin N (**1**)

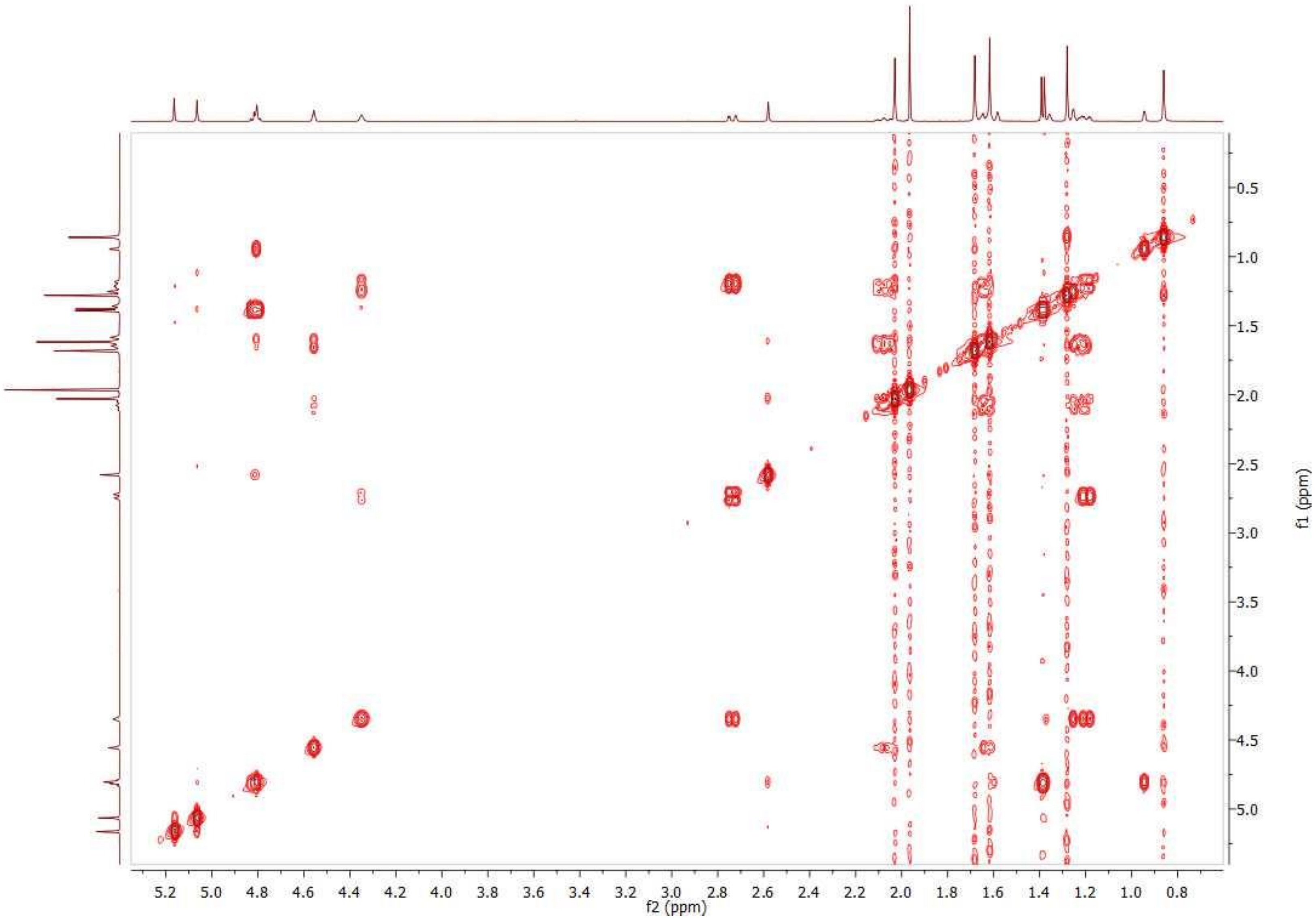

Figure S9: H,H-COSY spectrum ($CDCl_3$, 500 MHz) of terretonin N (**1**)

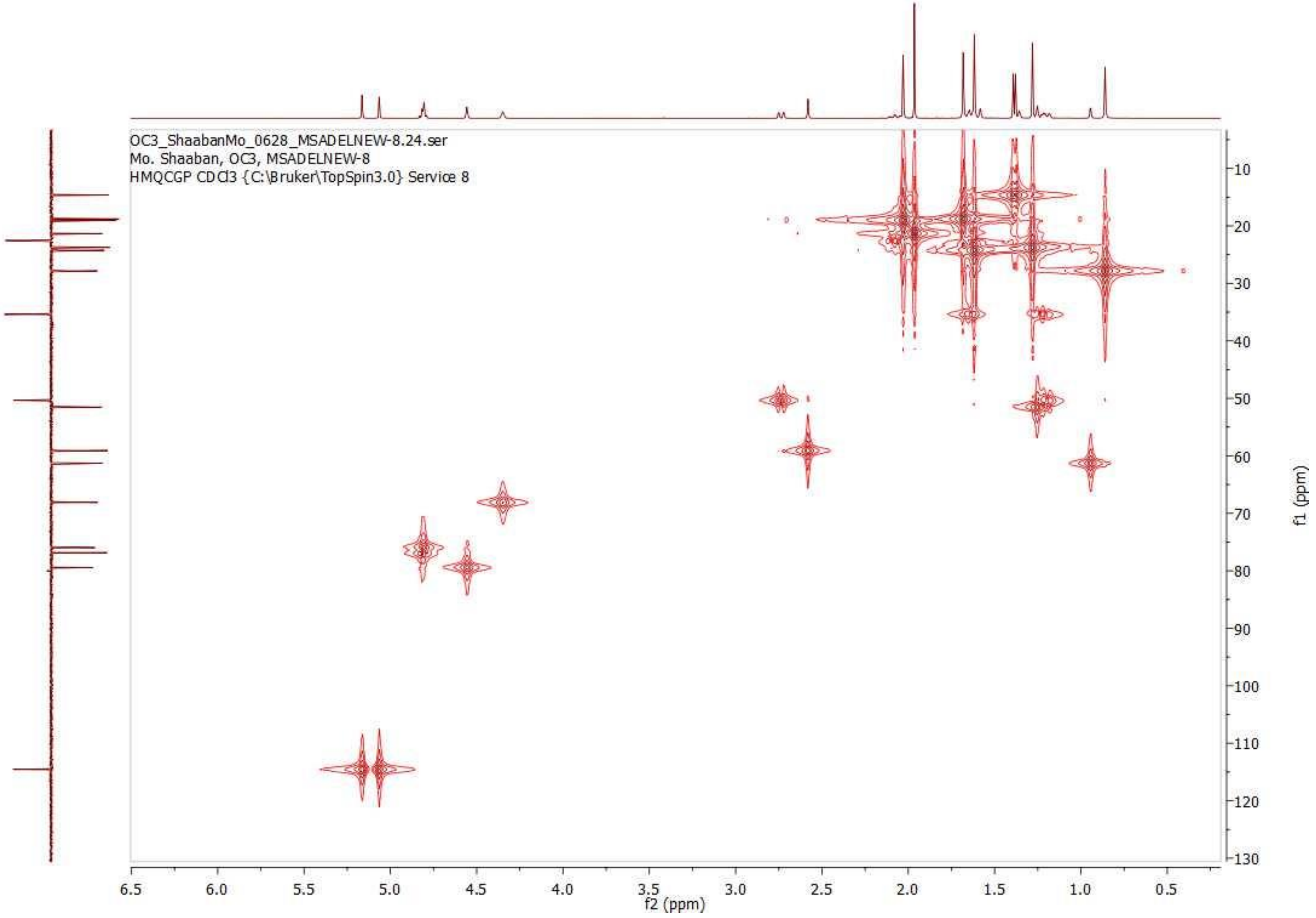

Figure S10: HMQC spectrum ($CDCl_3$, 500 MHz) of terretonin N (**1**)

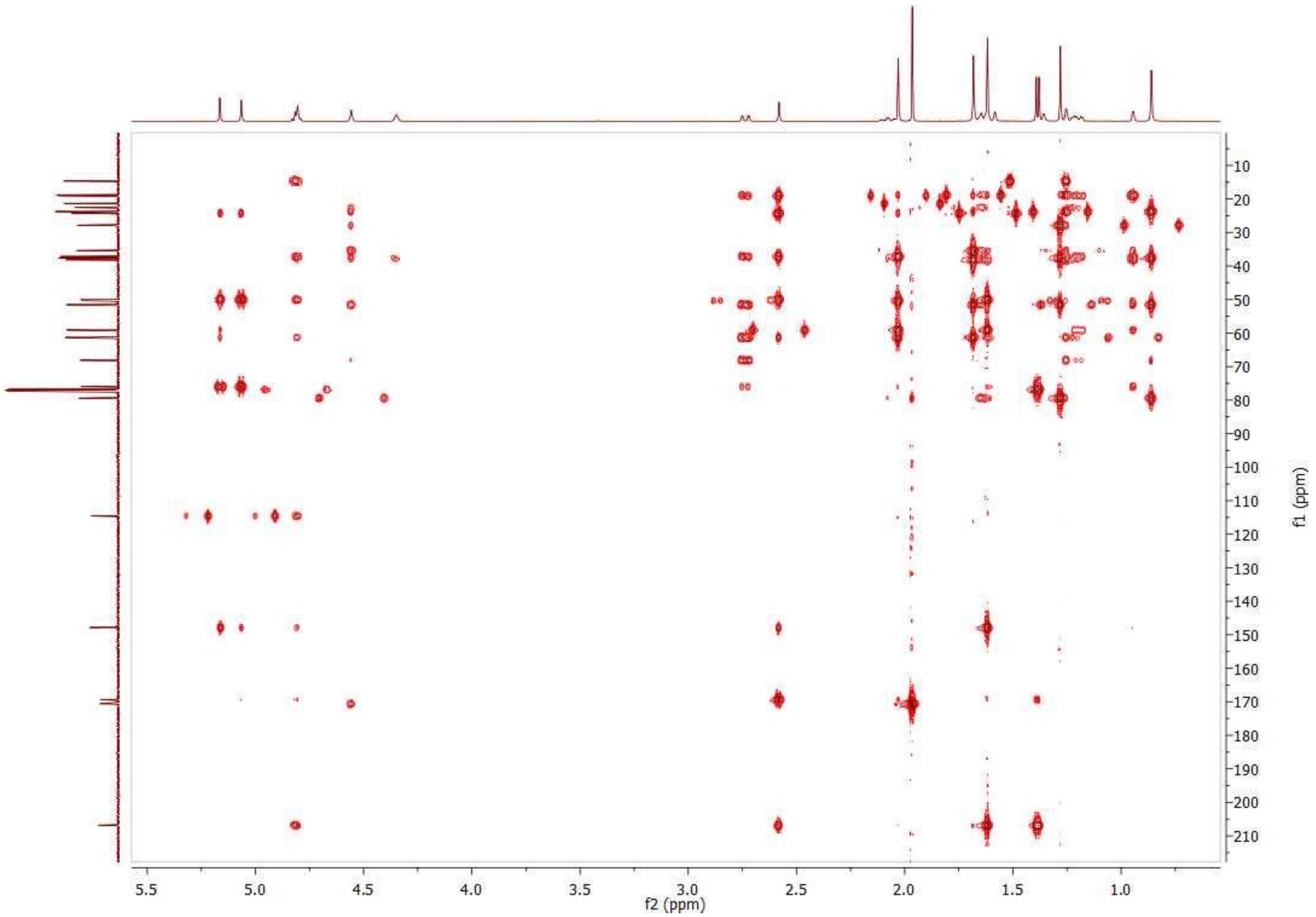

Figure S11: HMBC spectrum ($CDCl_3$, 500 MHz) of terretonin N (**1**)

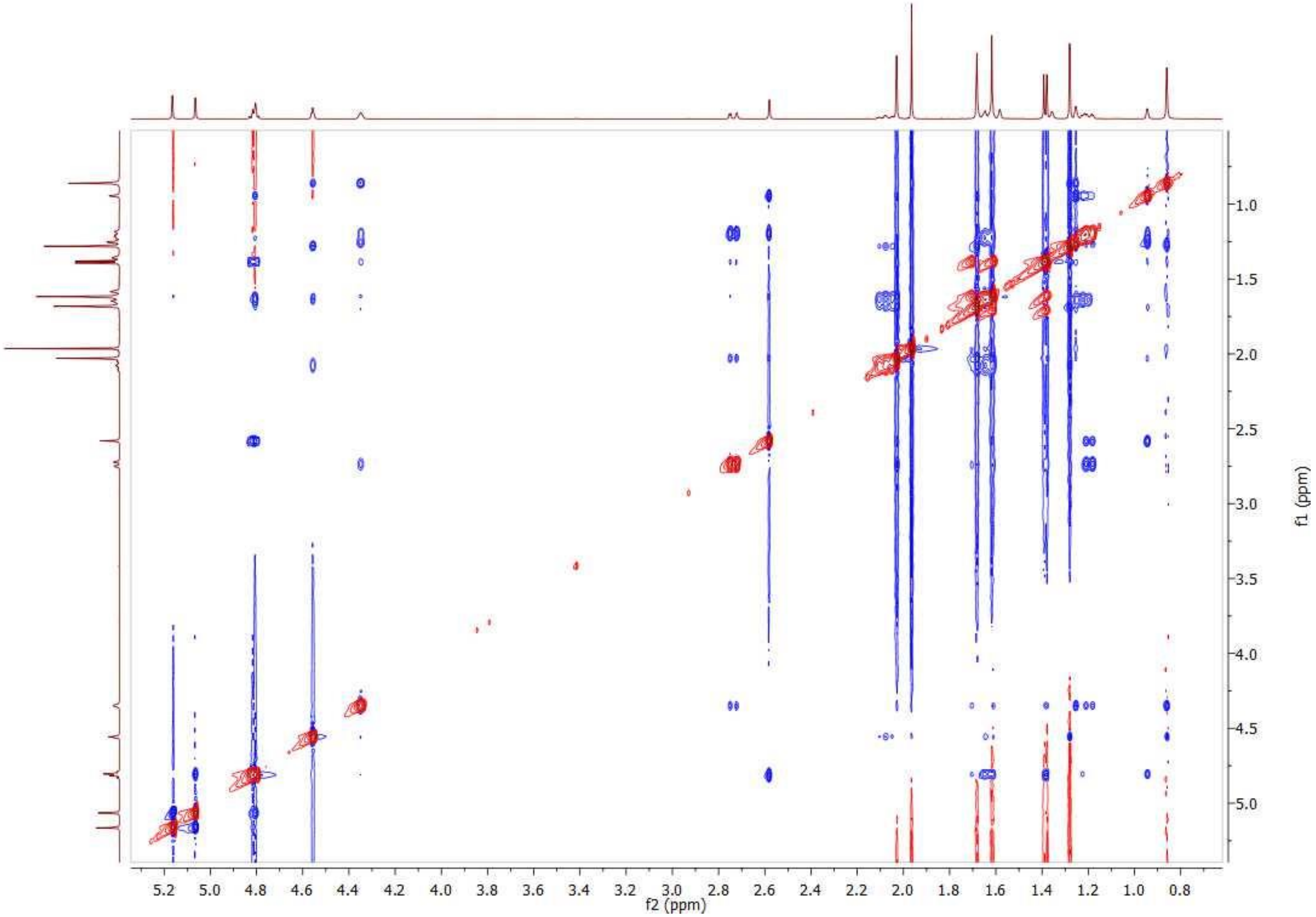

Figure S12: NOESYspectrum ($CDCl_3$, 500 MHz) of terretonin N (**1**)
